# Multilocus microsatellite typing reveals a genetic relationship but, also, genetic differences between Indian strains of *Leishmania tropica* causing cutaneous leishmaniasis and those causing visceral leishmaniasis

**DOI:** 10.1186/1756-3305-7-123

**Published:** 2014-03-25

**Authors:** Lena Krayter, Ram A Bumb, Kifaya Azmi, Julia Wuttke, Mariam D Malik, Lionel F Schnur, Poonam Salotra, Gabriele Schönian

**Affiliations:** 1 Institute of Microbiology and Hygiene, Charité-University Medicine Berlin, Hindenburgdamm 30, 12203 Berlin, Germany; 2 Department of Dermatology, SP Medical College, Bikaner, India; 3 Al-Quds Nutrition and Health Research Institute, Faculty of Medicine, Al-Quds University, Abu-Deis, P.O. Box 20760, West Bank, Palestine; 4 Department of Parasitology, The Kuvin Center for the Study of Infectious and Tropical Diseases, Hebrew University-Hadassah Medical School, Jerusalem, Israel; 5 National Institute of Pathology, Indian Council of Medical Research, Safdarjung Hospital Campus, New Delhi, India

## Abstract

**Background:**

Leishmaniases are divided into cutaneous (CL) and visceral leishmaniasis (VL). In the Old World, CL is caused by *Leishmania* (*L*.) *major*, *L. tropica* and *L. aethiopica. L. tropica* can also visceralize and cause VL. In India, the large epidemics of VL are caused by *L. donovani* and cases of CL are caused by *L. major* and *L. tropica*. However, strains of *L. tropica* have also been isolated from Indian cases of VL.

This study was done to see if Indian strains of *L. tropica* isolated from human cases of CL are genetically identical to or different from Indian strains of *L. tropica* isolated from human cases of VL and to see if any genetic differences found correlated with clinical outcome presenting as either CL or VL.

**Methods:**

Multilocus microsatellite typing (MLMT), employing 12 independent genetic markers specific to *L. tropica*, was used to characterize and identify eight strains of *L. tropica* isolated from human cases of CL examined in clinics in Bikaner City, Rajasthan State, north-west India. Their microsatellite profiles were compared to those of 156 previously typed strains of *L. tropica* from various geographical locations that were isolated from human cases of CL and VL, hyraxes and sand fly vectors.

**Results:**

Bayesian, distance-based and factorial correspondence analyses revealed two confirmed populations: India/Asia and Israel/Palestine that subdivided, respectively, into two and three subpopulations. A third population, Africa/Galilee, as proposed by Bayesian analysis was not supported by the other applied methods. The strains of *L. tropica* from Bikaner isolated from human cases of CL fell into one of the subpopulations in the population India/Asia together with strains from other Asian foci. Indian strains isolated from human cases of VL fell into the same sub-population but were not genetically identical to the Bikaner strains of *L. tropica*.

**Conclusions:**

It seems that the genetic diversity encountered between the two groups of Indian strains is mainly owing to their geographical origins rather than their different times of isolation. Also, the genetic differences seen between the dermatotropic and viscerotropic strains might be connected with the difference in pathogenicity.

## Background

The leishmaniases are endemic to 98 countries in the world. With 278,000 counted cases they are one of the most neglected tropical diseases. Alvar *et al*. [1] estimate the incidence to be much higher with 0.9 to 1.6 million cases per year. Annually, 20,000-40,000 patients die of the disease caused by the more pathogenic species of *Leishmania*. The leishmaniases have been divided into cutaneous (CL) and visceral leishmaniases (VL), and each of the two basic types of clinical conditions has its particular causative agents. In the Old World, CL is caused by *Leishmania major*, *L. tropica* and *L. aethiopica*, and VL by *L. donovani* and *L. infantum*.

However, it has long since been recorded that *L. donovani* and *L. infantum* can also cause CL without patent signs of visceral disease; bearing in mind that post-kala azar dermal leishmaniasis (PKDL) is a sequel to VL and not just a form of CL, and *L. tropica* can also visceralize and cause VL without patent cutaneous symptoms [2,3]. This has been confirmed by later studies [4-6].

In India, human VL caused by *L. donovani*, is of major concern since more than 100,000 cases are said to occur annually, most of them in Bihar State, north-eastern India [7]. Human CL has also been reported from different parts of India, e. g. Rajasthan State, north-west India [8], Himachal Pradesh, northern India [9], and Kerala State, south-west India [10]. Three species of *Leishmania* have been found to cause CL in India: *L. major*, *L. tropica* and *L. donovani*[1]. Bikaner City and its environment in Rajasthan State, which has a hot and dry climate, has been the main focus of CL in India [8] where the first case was reported in 1971 [11] and where the causative agent was shown to be a strain of *L. tropica* and the vector of the sand fly species *Phlebotomus* (*P*.) (*Paraphlebotomus*) *sergenti*[12,13]. *P. sergenti* is ubiquitous and the main vector for *L. tropica*. A strain of *L. tropica* was also isolated from a dog, the 'Bikaner dog strain' MCAN/IN/1971/DBKM [14,15]. Aara *et al*. [16] have summarized the cases of CL reported in Bikaner over the past decade. After a period of increasing numbers of infections from 2001 to 2009, there was a decrease in 2010 and 2011, and *L. tropica* was identified as the species causing the cases. Interestingly, Sharma *et al*. [9] saw cases of CL caused by *L. tropica* and, also, *L. donovani* among 161 cases of CL from a new epidemic focus in Himachal Pradesh, northern India. In the same focus, they also found a case of VL caused by *L. tropica*[17]. More recently, a case of VL from Kolkata caused by *L. tropica* was described [18,19]. Several cases of CL have been reported from Kerala, south-west India, but the identity of the parasite causing them is unknown [10]. As sand flies collected in the same area were identified as *P. argentipes*, which is considered to be the vector of *L. donovani* in India, one might surmise that these cases of CL were caused by *L. donovani*. In Sri Lanka, strains isolated from human cases of CL were identified as *L. donovani*[20]. Sri Lanka is geographically not far from Kerala State but a sea, part of the Indian Ocean, separates them.

It has generally been thought that where species of *Leishmania* are associated with more than one clinical condition, forming a clinical syndrome, e. g., *L. tropica* being associated with simple CL, leishmaniasis recidivans (LR) and VL, *L. donovani* with VL, CL, and oro-nasal leishmaniasis, *L. aethiopica* with CL, diffuse cutaneous leishmaniasis (DCL) and rare cases of mucocutaneous leishmaniasis (MCL), and *L. braziliensis* with CL and MCL, the human hosts are considered to be the varying part of the host-parasite relationship where the hosts' immunological status determines the clinical outcome and the parasites are considered a uniform unvarying entity. However, various means, including serological, biochemical and molecular genetic methods have shown that the different strains constituting the different leishmanial species vary, of course, among the species and also among the strains within each species, the strains of the species *L. tropica* particularly so [21-23].

The interest in this study was to see if Indian strains of *L. tropica* isolated from human cases of CL are genetically identical to or different from Indian strains of *L. tropica* isolated from human cases of VL; and, if they are different, how different. In doing this, the Indian strains were compared to other strains of *L. tropica* from many other places, isolated from cases of CL and VL from those places.

Microsatellite markers are co-dominant, highly variable and enable differentiation of strains at an intra-specific level. Therefore, multilocus microsatellite typing (MLMT) was applied to determining, firstly, the microsatellite profiles of Indian strains of *L. tropica* isolated from human cases of CL, secondly, their own genetic interrelationship and, then, their genetic relationship to Indian strains of *L. tropica* isolated from human cases of VL and many other strains of *L. tropica* from human cases and some from sand flies and animal hosts (Additional file 1: Table S1) to see whether genetic differences underlying different genotypes correlate with the different human clinical conditions.

Serological [21-23], biochemical [15,24], and molecular genetic [25] studies have shown that the species *L. tropica* is intrinsically very variable. Extensive genetic variation was confirmed by a study that investigated variation in 21 microsatellite markers of 117 strains of *L. tropica* from different African and Asian foci that identified ten different genetic groups [26]. In that study, four Indian strains of *L. tropica* isolated from cases of VL from Bihar State grouped in one cluster together with other strains from the Asian continent.

## Methods

### Ethical clearance

This study was approved by the Ethics Committees of the “S.P. Medical College and associated group of hospitals” in Bikaner Rajasthan State, India.

### Parasite strains

The eight strains of *L. tropica* typed genetically by MLMT were isolated in 2006 and 2007 from human cases of CL presenting at clinics in the City of Bikaner, Rajasthan State, north-west India. Their WHO codes are given in Additional file 1: Table S1.

MLMT genotypes and genetic population affiliations of 156 strains of *L. tropica* of widely distributed geographical origins, most of which were isolated from human cases of CL, some of which were isolated from human cases of VL and a few of which were isolated from sand fly vectors and hyrax hosts, are also given in Additional file 1: Table S1 for making comparisons. Those data comprise our own, as yet unpublished data and data published previously [26]. The strain MHOM/PS/2001/ISL590, whose microsatellite loci had been sequenced [27], was used as reference strain to compare the results of different PCR and fragment analysis runs.

### Microsatellite typing

The number of markers for the species *L. tropica* used in a previous study [26] was reduced for this one from 21 to 12, 10 of which are listed by Schwenkenbecher *et al*. [27] and Jamjoom *et al*. [28]. Furthermore, the primers for microsatellite marker GA9, also listed by Schwenkenbecher *et al*. [27], were modified and named as the new marker GA9n. Also, all new un typed and previously typed strains were typed, using a new microsatellite marker, 27GTGn (Table 1).

**Table 1:** Microsatellite markers used in this study

**m/sat**	**fw primer(5´→3´)**	**rev primer(5´→3´)**	**AT (°C)**	Fragment size(bp)*	Repeat array*
GA1	TCGGAGTCACCTCGCACCGC	GGTGGGGCAGGTAAAGCGGC	56	66	(GA)11
GA2	GATCACAGCGACGTCTGAAG	CCTGCTGCCACCATCTTAAGC	56	62	(GA)8
GA6	GTGTGAGCTAATCGATTGGG	CGCTCTCTCTGTCTCTGTCT	42	61	(GA)8
GA9n	CAAGTCCAAATCAGAAGAGC	CTCTATCCACTGCGTTTCTC	60	112	(GA)7
LIST7010	CGGTGAATGCCTAAAGAGAGA	AGGAACGCATACTTGGAAGG	42	190	(TA)28
LIST7011	CGGCGACATGCACACATA	CACACACATTGAAGATGGAGGA	42	186	(TA)15
LIST7027	CTCTCTCGTCACCACAGCAC	AGGGGACAAGACACAGATGG	50	181	(CA)12
LIST7033	CATTGCTGAGTGCTGCTAGTG	ATGAGCGTACTGGGCACAC	44	180	(GT)8
LIST7039	CTCGCACTCTTTCGCTCTTT	GAGACGAGAGGAACGGAAAA	44	205	(CA)16
LIST7040	GCAGAGCGAGACACACAGAC	GTGCACGTTGATGTGCTTCT	50	245	(GT)23
4GTG	CGGTTTGGCGCTGAAAGCGG	CGTGAGGACGCCACCGAGGC	58	62	(GTG)5
27GTGn	GATAGCGTTGGAGGCAAGC	CTATCCGCACCACGATCC	60	106	(GTG)5

AT, annealing temperatures in °C; *, fragment sizes and repeat arrays as in the reference strain MHOM/PS/2001/ISL590.

Each PCR reaction was done in a volume of 25 μl that contained 1× PCR buffer, 200 μM dNTP mix, 5 pmol fluorescence-labelled forward and unlabelled reverse primer, 1 unit Taq polymerase (Roche, Germany), and 20 ng genomic DNA. PCRs started with denaturation for 5 min at 95°C, followed by 35 cycles (30 sec at 95°C, 30 sec at marker-specific annealing temperature (AT, see Table 1), and 1 min at 72°C) of amplification. Reactions ended with a final extension step of 6 min at 72°C. The labelled primers were used to detect the fragments with an ABI sequencer and determine their sizes.

The GeneMapper software version 3.7 (Applied Biosystems, Foster City, USA) was used for the subsequent analysis of the peaks. The resulting microsatellite profiles were summarized in an excel file. Strains missing data for more than two microsatellite markers were excluded from the analyses and, thus, are not listed in Additional file 1: Table S1, leaving seven with 20 data points, i.e., two values per microsatellite marker, 28 strains with 22 values, and 129 with a complete set of data. For homozygous loci, the fragment length was used for both alleles. When two peaks appeared in the fragment analysis, the strain was considered to be heterozygous for that marker and both alleles were used for further analysis.

Fragment lengths had to be normalized for several reasons. Two different sequencers and different fluorescence dyes were used in each of two studies, this one and a previous one [26], which used the microsatellite profiles employed in this analysis. This altered the resulting fragment lengths by up to 2 bp. Also, the primer sequences for the marker GA9n were changed, which enlarged the resulting fragment. Because of this and also to identify differences in fragment sizes owing to different PCR runs and fragment analyses, the DNA of strain MHOM/PS/2001/ISL590 was included in each run as a standard reference for size. The data were normalized by calculating the repeat numbers and multiplying them by the size of the di- or tri-nucleotide microsatellite repeat, and finally adding the size of the flanking region.

Variation in the 12 independent microsatellite markers in the eight strains of *L. tropica* from Bikaner was determined and compared with the microsatellite profiles of the 156 strains typed previously.

### Population genetic analyses

Once the input file was created with MSA version 4.05 [29], the Bayesian clustering approach implemented in STRUCTURE software version 2.3.4 [30] was used to investigate the population structure. This program identifies genetically distinct groups by analysis of allele frequencies and estimates the group membership coefficient for each individual. The length of the Burnin Period was set to 20000 and the number of Markov Chain Monte Carlo Repeats after Burnin to 200000. Calculation of ∆K, as described in [31], revealed the most likely number of populations for 10 replicate runs for each K.

As an additional statistical approach based on allele frequencies, the data were processed by factorial correspondence analysis (FCA) as implemented in Genetix 4.05 [32]. The conversion of the input file for FCA was done using CONVERT 1.31 [33].

POPULATIONS software version 1.2.34 (http://bioinformatics.org/~tryphon/populations/) was applied to calculate the genetic distances, using the shared alleles distance measure (DAS). The Neighbour Joining (NJ) tree based on these data was visualized by MEGA 5.1 [34].

The algorithm of SplitsTree 4.12.8 [35] was applied for creating a phylogenetic network, which accounts for reticulation events such as hybridization, horizontal gene transfer or recombination.

Calculations of *F*_ST_ values (genetic distances between populations), and of mean number of alleles (A), observed (*H*_o_), expected (*H*_e_) heterozygosity, and the inbreeding coefficient (*F*_IS_) were performed with MSA 4.05 and GDA 1.1 [36], respectively.

## Results

The whole set of samples comprised 97 different genotypes, of which 75 were unique to individual strains within the set. Six of the eight strains from Bikaner, BKC-1; -2; -10; -11; -15; -28, were of identical genotype, one, BCK-3 differed from the others in a single microsatellite marker, LIST7040, and in one strain, BCK-5, amplification failed for one marker, GA1.

The eight Indian strains isolated from cases of CL from Bikaner showed less variation among their microsatellite profiles (Additional file 1: Table S1, genotypes *Ltro*MS 030, 031, 032) when compared to those of the four Indian strains isolated from cases of VL between 1979 and 1997 (Additional file 1: Table S1, genotypes *Ltro*MS 004, 017, 018). Compared with the eight Indian strains isolated from cases of CL from Bikaner, the DNA from all four older Indian strains isolated from cases of VL showed different repeat lengths in three of the microsatellite markers, GA2, LIST7033, and LIST7040. Four other microsatellite markers, GA9n, LIST7010, LIST7027, and LIST7039, were different in at least one of the strains (Additional file 1: Table S1). Apart from the Indian strains, among the whole set of strains, there were three other big groups, in each of which the strains were of the same genotype. Two of the three groups contained Turkish strains, one of which was twelve strains of genotype *Ltro*MS 027 and the other of which was nine strains of genotype *Ltro*MS 028. Their only difference was the failure to amplify one marker, 27GTGn. The other big group contained 26 Israeli and Palestinian strains of genotype *Ltro*MS 041 (Additional file 1: Table S1).

The different algorithms implemented through various computer software programmes to analyse the genetic structure of the strains of *L. tropica* collected in various regions endemic to the leishmaniases differ in the way they analyse microsatellite data. By using different approaches, one can evaluate whether the different algorithms used do or do not support each other.

**Figure 1:**
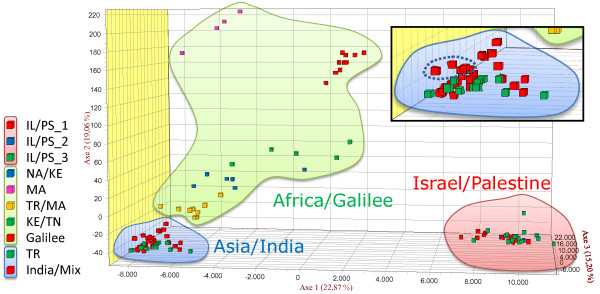
**Factorial correspondence analysis.** Phylogenetic relationship between the populations of *L. tropica* calculated by Factorial Correspondence Analysis (FCA). The Bikaner strains are circumscribed by a dotted line. Bayesian results are indicated by colours: background colours show main populations, coloured squares represent subcomponents within populations. The strains, one representative for each genotype, were assigned to the proposed populations before applying FCA.

**Figure 2:**
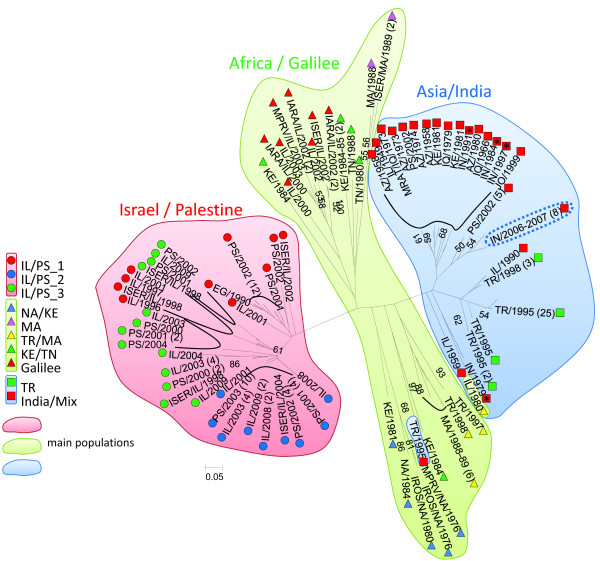
**Neighbour joining tree.** Phylogenetic relationship between strains of *L. tropica* based on the proportion of shared alleles. Bootstrap values > 50 are indicated at the nodes. Strain labels specify: the animal host or sand fly vector by their conventional four letter code; the country of origin by the conventional two letter code; and the year of isolation. The codes for sand fly vectors, animal hosts, and countries are given in Additional file 1. If not indicated otherwise, strains were from human cases. The Bikaner strains are circumscribed by a dotted line. Strains from human cases of VL from India are indicated by an asterisk. Strains of the same identity share a single label with the number of identical strains specified in parentheses. Bayesian results are indicated by colours: background colours show main populations; coloured squares, triangles and circles represent subcomponents within populations.

**Figure 3:**
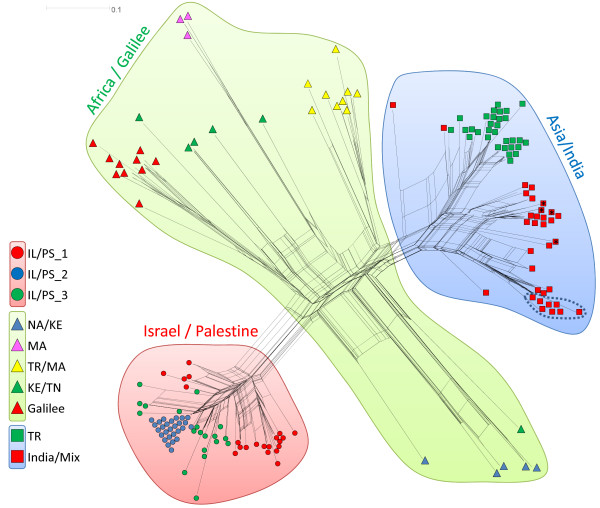
**Neighbour network.** NeighbourNet presenting the genetic relationship between strains of *L. tropica* as calculated by SplitsTree 4. Cross connections denote probable reticulation events between strains like hybridisation, recombination, or horizontal gene transfer. The Bikaner strains are circumscribed by a dotted line. Strains isolated from human VL patients from India are indicated by an asterisk. Bayesian results are indicated by colours: background colours show main populations; coloured squares, triangles and circles represent subcomponents within populations.

STRUCTURE analysis, which is based on Bayesian statistics and uses allele frequencies to study the nature and extent of genetic variation within and between populations, followed by ∆K calculation, which determines the most probable number of populations, revealed three main populations in the whole set of data that correlated largely with strains´ geographical origins: Asia/India, Israel/Palestine and Africa/Galilee. In Figures 1, 2 and 3, the coloured clouds and geometrical symbols indicate the main genetic populations and their subpopulations respectively, and reflect the assignment of the strains to genetic groups according to Bayesian statistics. The Indian strains isolated from cases of CL from Bikaner grouped together with the older Indian strains isolated from cases of VL from Bihar State and one additional strain from a case of VL from India, in the population Asia/India, which also contained strains from many Middle Eastern and other Asian foci, making a total of 64 strains. Here, 32 Turkish strains, of which 28 were isolated during a local outbreak near Sanliurfa, formed a distinct group. A second genetic subpopulation, designated India/Mix, in the population Asia/India comprised strains from different geographical locations, many of which shared an early time point of isolation. The second population, designated Israel/Palestine, as it contained 66 strains from Israel and Palestine and one from Egypt, separated into three genetic subpopulations that did not correlate with the geographical distribution of the locations from which the strains come from. The third population, designated Africa/Galilee comprised 33 strains, 23 strains from various parts of Africa, from its northern border to its southern one, and ten strains from one restricted focus north of the Sea of Galilee in Israel. The population Africa/Galilee separated into five genetic subpopulations. One subpopulation consisted of the ten strains from Galilee. The other four were given designations according to the geographical origins of the strains in them (KE/TN, NA/KE, TR/MA, and MA). As the subdivisions within the main populations were not well supported by the ∆K values (Additional file 2: Figure S1), sub-clustering was carefully re-evaluated using the other analytical methods described.

FCA is a multidimensional statistical method that, like STRUCTURE, uses allele frequencies for its calculations. This approach clearly confirmed the two main populations Asia/India and Israel/Palestine identified by STRUCTURE (Figure 1). Again, the strains from Bikaner grouped together with other Asian strains. However, the strains of the third main population that was discerned by STRUCTURE, Africa/Galilee, were widely dispersed when FCA was applied. In this population separation at the subpopulation level was seen, but to a much lesser extent. The strains from the focus north of the Sea of Galilee were still clearly separated from the other strains. Also the two groups containing Moroccan strains (MA and TR/MA) could qualify as clusters.

In contrast to Bayesian statistics and FCA, distance-based software like populations and SplitsTree 4 uses the proportions of shared alleles for their analyses.

In the NJ tree resulting from calculating genetic distances (Figure 2) the eight Bikaner strains from human cases of CL clustered together with the other Asian strains, forming a big monophyletic group. However, they formed a separate branch within this cluster whereas three of the four Indian strains from human cases of VL grouped together with the older strains of different geographical origins on another branch. These three strains from human cases of VL all were from Bihar state in India. The fourth Indian strain from a human case of VL (IN/1979) of unknown geographical origin did not cluster with the other Indian strains. Once again, the Turkish strains from the outbreak of CL that occurred in the vicinity of Sanliurfa formed a distinct group. In this analysis, the population Africa/Galilee appeared as a paraphyletic group. However, the strains from the focus north of the Sea of Galilee and some strains from Kenya, Tunisia, and Morocco, grouped together as a monophyletic branch within the paraphyletic group.

The neighbour network picture (Figure 3) resembled that of the NJ tree (Figure 2). Again, the eight Bikaner strains from human cases of CL were located near but not very close to the Indian strains from human cases of VL from Bihar State. The Israeli and Palestinian strains clustered together in one big genetic group. In the third population, Africa/Galilee, three clear subpopulations were seen, the Galilee group and two different groups of Moroccan strains. All the other strains in this population were dispersed showing no clear pattern.

The mean fixation index (*F*_ST_) values between the populations ranged from 0.30 to 0.65 (Additional file 3: Table S2). The smaller the value, the smaller the genetic distance between the two groups, with *F*_ST_ = 0 meaning complete identity and *F*_ST_ = 1 indicating complete separation. Values greater than 0.25 indicate a very large genetic difference between populations as was found for the three main populations discerned by this study. The *F*_ST_ value for the Turkish and the subpopulations within the population Asia/India was 0.36, confirming that these two groups are clearly separate, as shown by the analyses above.

The number of alleles per locus ranged from 3 (GA6, GA9n, and 4GTG) to 16 (LIST7039), with an average of 7.42 (Table 2). The expected heterozygosity (*H*_e_), ranging from 0.06 to 0.814, was generally higher than the observed heterozygosity (*H*_o_), ranging from 0.000 to 0.402. The inbreeding coefficients per locus (*F*_IS_) showed more positive than negative values, indicating a large number of homozygotes among these strains. The *F*_IS_ value of the population Asia/India, 0.091, indicated nearly random mating (Table 3). The *F*_IS_ value of the population Africa/Galilee, 0.936, indicated almost complete inbreeding.

**Table 2:** Descriptive analyses per locus

**Locus**	**A**	***H***_**o**_	***H***_**e**_	***F***_**IS**_
**GA1**	4	0.012	0.060	0.786
**GA2**	9	0.104	0.677	0.667
**GA6**	3	0.000	0.161	1.000
**GA9n**	3	0.393	0.495	-0.255
**LIST7010**	10	0.157	0.814	0.752
**LIST7011**	8	0.006	0.738	0.983
**LIST7027**	10	0.402	0.808	0.260
**LIST7033**	7	0.006	0.609	0.979
**LIST7039**	16	0.116	0.798	0.782
**LIST7040**	11	0.358	0.660	-0.023
**4GTG**	3	0.000	0.192	1.000
**27GTGn**	5	0.248	0.664	0.118
**all**	7.42	0.150	0.556	0.543

A, number of alleles; *H*_o_, observed heterozygosity; *H*_e_, expected heterozygosity; *F*_IS_, inbreeding coefficient.

**Table 3:** Descriptive analyses per population

**Population**	**A**	***H***_**o**_	***H***_**e**_	***F***_**IS**_
**Asia/India**	3.08	0.340	0.358	0.091
**Africa/Galilee**	5.92	0.050	0.669	0.936
**Israel/Palestine**	2.42	0.030	0.140	0.763

A, number of alleles; *H*_o_, observed heterozygosity; *H*_e_, expected heterozygosity; *F*_IS_, inbreeding coefficient.

The overall outcome of combining the results of the various analytical strategies, i.e. the existence of two distinct populations among the strains of *L. tropica*, India/Mix and Israel/Palestine, was confirmed by all the algorithms used. However, the third population, Africa/Galilee, suggested by Bayesian statistics, was not confirmed by the other algorithms.

## Discussion

MLMT of the Indian strains of *L. tropica* collected in Bikaner, Rajasthan State, in 2006 and 2007 from human cases of CL showed them to be more closely related to strains of *L. tropica* from various Asian geographical origins than to those from non-Asian origins. MLMT and subsequent analysis assigned them to the same monophyletic cluster of population Asia/India as three older Indian strains isolated from human cases of VL collected in Bihar State, albeit clearly separated from them. There are several possible reasons for the divergence of these two groups of Indian strains. One possibility is geographical origin as shown in several other microsatellite typing studies on different species of *Leishmania*[37-39]. The eight new Indian strains isolated from human cases of CL came from Bikaner located in Rajasthan State, which is in north-western India whereas three of the four old Indian strains isolated from human cases of VL came from Bihar State, which is in north-eastern India, with a vast geographical distance between the two states. Since there are strains isolated from human cases of VL from other, even more distant countries, namely Israel, Iraq, Kenya, in the same monophyletic group, geographical separation is probably not the only or not the most important explanation for these findings. A second possibility for the separation is the time difference between the isolation of the strains from cases of CL and the isolation of those from cases of VL. The effect of passage of time was shown in previous studies on leishmanial strains in the *L. donovani* complex [37] and on strains of *L. tropica*[26]. The time between the isolation of the two groups of Indian strains varied as the strains from the cases of VL were isolated at different times, giving a span of 10, 16, 23, and 28 years, depending on the visceralizing strain (Additional file 1: Table S1). The long period of time, possibly, much of which the strains have been grown in artificial culture media, might have permitted or caused genetic mutations to occur. Almost all the strains from this monophyletic group were isolated at early time points. Considering this, not only the different place but also the more ancient time of isolation of the strains might be correlated to the genotype differences inferred by microsatellite typing. A third possibility is the existence of longstanding intrinsic genetic differences between the two types of leishmanial parasites, causing one type to be dermatotropic, the other to be viscerotropic and dictating the difference in the style of their pathogenicity, leading to VL in the case of the older Indian strains and CL in the case of the more recently isolated Bikaner strains. However, previous studies on microsatellite variations among different species of *Leishmania* failed to confirm such a genotype-phenotype association [38,40]. Moreover, there is overwhelming evidence on the effect of human hosts’ nutritional and immunological status on the development and style of the various leishmaniases [41-43].

The strain from India collected in 1979 was found on a separate branch of the population Asia/India. Since the origin of this strain is not known, geographic separation might be a possible explanation for this as well as the effect of numerous passages of the culture.

Of the four non-Indian strains isolated from cases of VL, one was Israeli, isolated in 1949 that typed as genotype *Ltro*MS 001, one was Iraqi, isolated in 1979 that typed as genotype *Ltro*MS 005, and two were Kenyan, isolated in 1981 that typed as genotypes *Ltro*MS 005 and *Ltro*MS 006. Their genetic similarity could be construed as being linked to the similar time of their isolation in the case of the last three strains. However, the first strain was isolated 30 and 40 years before the other three strains. Also, many other old strains isolated from human cases of CL from geographically widely distributed foci fall into the same subpopulation of the population Asia/India (Figure 2).

Further studies should include more strains of *L. tropica* recently isolated from human VL and CL cases in different parts of India to prove our hypotheses. It would be especially interesting to analyse strains from the focus in Himachal Pradesh where *L. tropica* was confirmed causing both, human CL and VL [9,17] to investigate if the genetics of the parasite plays a role in determining the style of a human case of leishmaniasis and whether it remains cutaneous or visceralizes.

The use of the reduced set of 12 microsatellite markers, largely confirmed the phylogenetic structure discerned previously for the species *L. tropica* by Schwenkenbecher *et al*., who used 21 microsatellite markers [26]. A more precise way of calculating ∆K was used here for determining the most probable number of populations using Bayesian statistics [31]. Three main genetic populations, Asia/India, Israel/Palestine and Africa/Galilee, were exposed that subdivided, respectively into two, three and five subpopulations. However, the subpopulations of the latter two populations could not be confirmed by the distance-based and networking methods. Despite these differences, which resulted from the different methods of calculation, the population structure presented here in general resembles the one produced by Schwenkenbecher *et al*. [26].

A few strains did not cluster according to their reported geographical origin. Three Israeli and five Palestinian strains isolated from human cases of CL were assigned to the population Asia/India. These discrepancies might be owing to those people getting infected with *L. tropica* while visiting regions in other countries where CL is endemic but being diagnosed in clinics after returning home and being registered as local cases; thus, importing foreign strains of *L. tropica*.

The African population suggested by Bayesian analysis was not confirmed by the other methods. The strains are too diverse in the correspondence analysis (Figure 1), the NJ tree (Figure 2) and the Network (Figure 3) to be regarded as a distinct population. For a clearer picture of the population structure in Africa, more strains from different African foci need to be investigated.

A group of strains isolated in Israel formed a distinct subpopulation within the essentially African population Africa/Galilee. They came from a focus on the northern side of the Sea of Galilee and were previously shown to be genetically [26] and antigenically [44] distinct from all the other Israeli strains of *L. tropica* studied. Significantly, the main vector in this focus is *P*. (*Adlerius*) *arabicus*[22,44] rather than *P*. (*Paraphlebotomus*) *sergenti*, which is the vector in the other Israeli foci [23] and, in fact, most of the geographical range of *L. tropica*. Also, it was in this focus that a strain of *L. tropica* was isolated from a hyrax of the species *Procavia capensis* and hyraxes are thought to be the animal reservoir of *L. tropica*[44]. That the Galilean strains cluster together with African strains might indicate the former´s African origin; they possibly having been brought into Israel by the hyraxes, as the species *P. capensis* is found all along the Great Rift Valley, which stretches from northern Syria via the Jordan Valley that separates Israel and Jordan then through East Africa to end in central Mozambique in southern Africa.

## Conclusions

Microsatellite typing was used to genotype eight Indian strains of *L. tropica* isolated from human cases of CL, which were examined at clinics in Bikaner City. The MLMT profiles of these strains showed genetic similarities to and, also, differences from those of older Indian strains of *L. tropica* isolated from human cases of VL. However, all the different analyses applied to these dermatotropic and the viscerotropic strains consigned them to the same main genetic population, together with strains of *L. tropica* from other Asian foci. It is assumed that the genetic diversity seen among the Indian strains of *L. tropica* examined is not only owing to their different geographical origins but also to their different times of isolation; however, we cannot exclude the possibility of genetic differences underlying the different genotypes of Indian strains of *L. tropica* being responsible for their difference in pathogenicity. This might be resolved if more strains of *L. tropica* from different foci of CL and VL would be included in the study, including those from the focus in Himachal Pradesh where cases of both CL and VL have been caused by *L. tropica*.

In comparing the Indian strains with the strains of *L. tropica* from other countries, three main genetic populations, Asia/India, Israel/Palestine and Africa/Galilee, were discerned, the strains of which separated into subpopulations with some geographical anomalies occurring. Genetic changes occur in a given place at a given time for whatever reason and spread in a population from that place during more time. One can assume that genotypes that appear to be out of geographical synchronization have been imported in some way. Animals, most insects and humans travel, including animal hosts and vectors. Humans are particularly well-travelled, more so today than ever before, and their parasites go with them wherever they go. Only where suitable hosts, animal or human, and vectors are available, zoonotic or anthroponotic leishmaniases will establish themselves.

## Competing interests

The authors declare that they have no competing interests.

## Authors’ contributions

LK carried out the microsatellite typing, conducted the data analysis, and wrote the manuscript. RAB collected the samples. KA, JW, and MDM carried out microsatellite typing. LS and PS isolated the sample DNA and participated in writing the manuscript. GS designed the study, participated in data analysis and writing the manuscript. All authors have read and approved the final manuscript.

## Supplementary Material

Additional file 1: Table S1 Strains used in this study. Doubled strains in the table represent different clones of the same strain received from different laboratories. In the genotype designations *Ltro* indicates *L. tropica* and MS microsatellite, in an attempt to initiate a standardized nomenclature and identification system for microsatellite profiles and their corresponding genotypes. Doc = documentation: *cited in Schwenkenbecher et al., 2006; **this study, ***own unpublished data. The fragment lengths are given in bp and were normalized to the reference strain MHOM/PS/2001/ISL590 as mentioned in the methods section. Click here for file

Additional file 2: Figure S1 Calculation of the most probable number of populations, ∆K, based on the results of Bayesian statistics. **A:** calculation for all strains in this study to specify the number of main populations, B-D: sub-structuring of the proposed populations (**B:** Asia/India, n = 64; **C:** Africa/Galilee, n = 33; **D:** Israel/Palestine, n = 67). Click here for file

Additional file 3: Table S2 *F*_ST_ values calculated for the three main populations identified by Bayesian analyses. *F*_ST_ estimates describe the genetic distances resulting from pairwise comparison of the distinct populations. Values are categorized into little (<0.05), moderate (0.05-0.15), great (0.15-0.25), and very great (>0.25) genetic differentiation. Click here for file
