# Supplementary figures and images for: Multilocus microsatellite typing reveals a genetic relationship but, also, genetic differences between Indian strains of Leishmania tropica causing cutaneous leishmaniasis and those causing visceral leishmaniasis
Source: Parasit Vectors. 2014 Mar 25;7:123. doi: 10.1186/1756-3305-7-123 (PMC3987047; doi:10.1186/1756-3305-7-123)

A

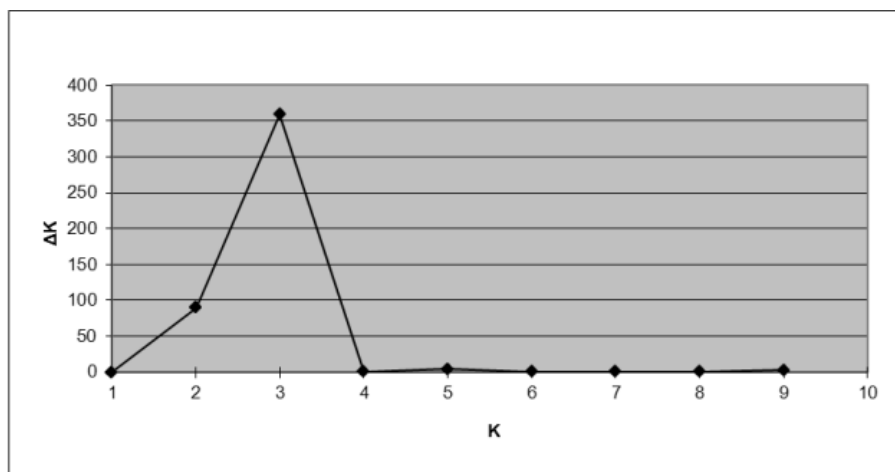

B

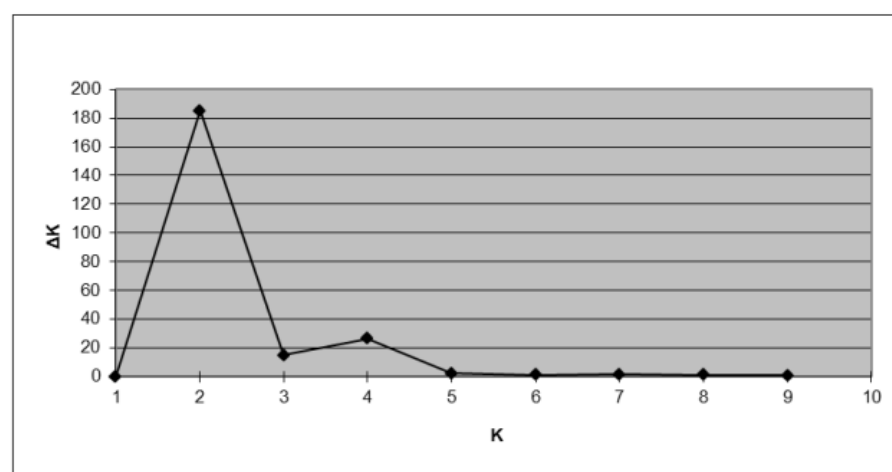

C

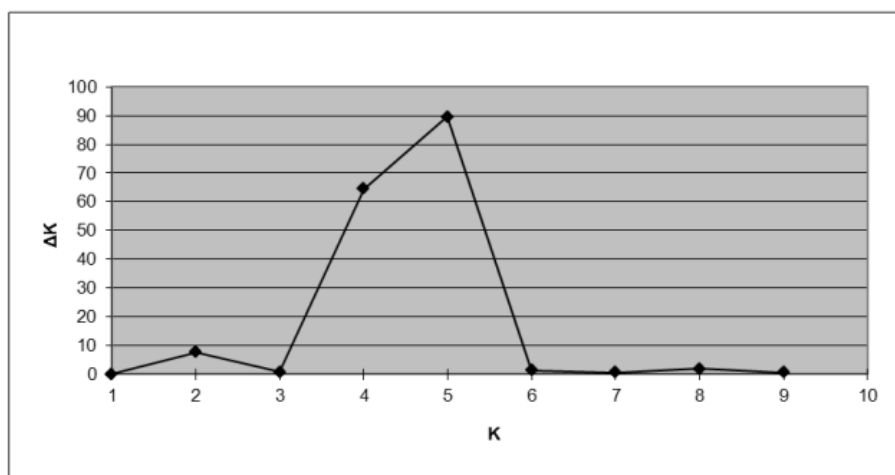

D

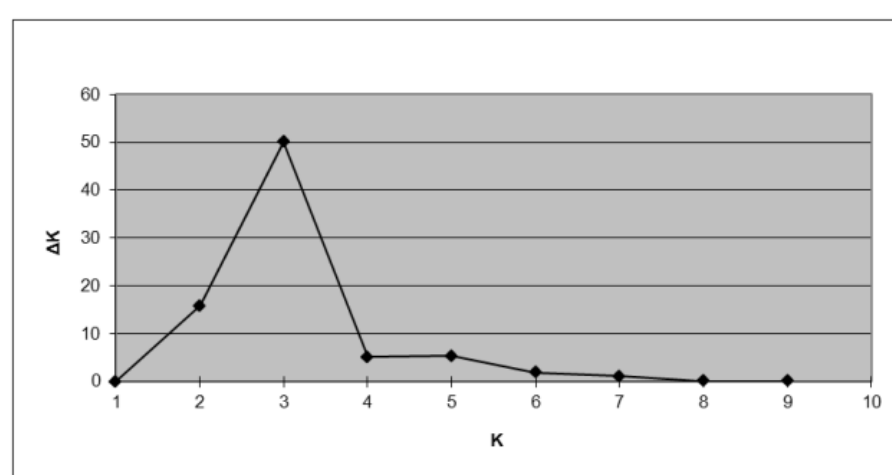

Supplement: Additional file 2: Figure S1 — Calculation of the most probable number of populations, ∆K, based on the results of Bayesian statistics. A: calculation for all strains in this study to specify the number of main populations, B-D: sub-structuring of the proposed populations (B: Asia/India, n = 64; C: Africa/Galilee, n = 33; D: Israel/Palestine, n = 67). [file 1756-3305-7-123-S2.pdf]
