# Supplementary material for: Multilocus microsatellite typing reveals a genetic relationship but, also, genetic differences between Indian strains of Leishmania tropica causing cutaneous leishmaniasis and those causing visceral leishmaniasis
Source: Parasit Vectors. 2014 Mar 25;7:123. doi: 10.1186/1756-3305-7-123 (PMC3987047; doi:10.1186/1756-3305-7-123)
Supplement: Additional file 3: Table S2 — FST values calculated for the three main populations identified by Bayesian analyses. FST estimates describe the genetic distances resulting from pairwise comparison of the distinct populations. Values are categorized into little (<0.05), moderate (0.05-0.15), great (0.15-0.25), and very great (>0.25) genetic differentiation. [file 1756-3305-7-123-S3.pdf]

A

| $F_{ST}$-values: | Africa/Galilee | Israel/Palestine | Asia/India |
|---|---|---|---|
| **Africa/Galilee** | 0.000000 | 0.489444 | 0.295047 |
| **Israel/Palestine** | 0.489444 | 0.000000 | 0.654935 |
| **Asia/India** | 0.295047 | 0.654935 | 0.000000 |

B

| $F_{ST}$-values: | Galilee | KE/TN | NA/KE | TR/MA | MA | IL/PS_1 | IL/PS_2 | IL/PS_3 | India/Mix | Sanliurfa |
|---|---|---|---|---|---|---|---|---|---|---|
| **Galilee** | 0.000000 | 0.425806 | 0.681216 | 0.606210 | 0.675700 | 0.711058 | 0.897599 | 0.731290 | 0.606826 | 0.749087 |
| **KE/TN** | 0.425806 | 0.000000 | 0.405547 | 0.436886 | 0.380469 | 0.563150 | 0.808112 | 0.576756 | 0.409833 | 0.617258 |
| **NA/KE** | 0.681216 | 0.405547 | 0.000000 | 0.571597 | 0.612644 | 0.730451 | 0.928371 | 0.746110 | 0.548077 | 0.673733 |
| **TR/MA** | 0.606210 | 0.436886 | 0.571597 | 0.000000 | 0.590991 | 0.671312 | 0.871756 | 0.690890 | 0.402785 | 0.555230 |
| **MA** | 0.675700 | 0.380469 | 0.612644 | 0.590991 | 0.000000 | 0.790676 | 0.967932 | 0.797866 | 0.580788 | 0.745751 |
| **IL/PS_1** | 0.711058 | 0.563150 | 0.730451 | 0.671312 | 0.790676 | 0.000000 | 0.586964 | 0.353305 | 0.612103 | 0.750701 |
| **IL/PS_2** | 0.897599 | 0.808112 | 0.928371 | 0.871756 | 0.967932 | 0.586964 | 0.000000 | 0.254642 | 0.728887 | 0.860386 |
| **IL/PS_3** | 0.731290 | 0.576756 | 0.746110 | 0.690890 | 0.797866 | 0.353305 | 0.254642 | 0.000000 | 0.628066 | 0.763749 |
| **India/Mix** | 0.606826 | 0.409833 | 0.548077 | 0.402785 | 0.580788 | 0.612103 | 0.728887 | 0.628066 | 0.000000 | 0.356349 |
| **Sanliurfa** | 0.749087 | 0.617258 | 0.673733 | 0.555230 | 0.745751 | 0.750701 | 0.860386 | 0.763749 | 0.356349 | 0.000000 |
